# Puerperal Fever, Complicated with Secondary Uterine Hemorrhage

**Published:** 1865-09

**Authors:** Swayne Wickersham

**Affiliations:** Chicago, Ill.


ARTICLE XXIX.

### PUERPERAL FEVER, COMPLICATED WITH SECONDARY UTERINE HEMORRHAGE.

BY SWAYNE WICKERSHAM, M.D., Chicago, Ill.

In hastily turning the pages of my note-book, I find no case that seems to me of greater interest than the one that I am about to bring to the attention of the members of the Medical Society. In its hemorrhagic feature, it may be regarded as somewhat unique. I have examined the medical literature of the subject with considerable care, and have come to the conclusion that if similar cases are of frequent occurrence, that clinical observers have but seldom recorded them. In order that an intelligent opinion may be formed, it becomes necessary that a brief history should be given of the case from the initial stage, which I will now proceed to do.

On the 7th of May, 1863, at 7 P.M., I was called to see Mrs. Evans, of this city. She was engaged in her fourth labor. I was then informed that the premonitory symptoms of the parturient effort had declared themselves about 8 A.M. From that hour, until about 4 P.M., she had but an occasional uterine pain, and a slight mucous discharge from the vagina. At the latter hour, the uterine contractions became much more frequent and expulsive in their character. When I was called, I found her cheerful and composed, and her labor apparently well developed. Her condition gave me no unusual solicitude. Her pains were moderately frequent, and of considerable force; her circulation and respiration were natural; her cutaneous surface was moist; her stomach was not irritable; there were no impacted fæces in the rectum; the bladder had just been voided of its contents; and the placental membranes had been ruptured half an hour previously. A vaginal examination was made, when there was found to be an abundant secretion of mucus, the os uteri widely dilated and retracted, the pelvis was amply capacious, and there was a complete absence of all rigidity of the adjacent tissues. The presentation was cephalic, and of the left occipito-acetabular position. I did not then regard the head as impacted. I detected no undue pressure upon the soft parts, consequently, after carefully considering all that I have stated, I expected a safe and speedy delivery.

At 11 P.M., four hours after I had been called to her, no perceptible progress had been made; the condition of the mother and child were not materially altered, except a slight increased action of the heart; the pains had been tolerably frequent and quite forcible. I was unable to account for the delay, and, as nothing had been accomplished during the interval by the natural effort, I was apprehensive that nature had done her best, and that exhaustion and subsidence of the pains would eventually supervene. I therefore recommended instrumental assistance.

Dr. Orrin Smith was called in council at 11½ P.M. He carefully investigated the case, and then concurred with me, that the forceps should be applied. The pulse was, at this time, evidently on the increase, and the pains much less active. The forceps were applied about midnight.

At 12½ A.M., of the 8th, there was almost a complete subsidence of the pains; the circulation had increased to 120, and the respiration in a corresponding ratio. Notwithstanding the administration of the tincture of camphor, gentle pressure over the uturus, and judicious traction of the forceps, I think there was an interval of thirty minutes between the last two pains, and these of but little force. No ergot was at hand.

The child was removed about 1 A.M. of the 8th, and was dead. The placenta was soon after removed, during a very feeble uterine contraction. The pulse was 140, and of but little volume; skin cold, and bedewed with a clammy perspiration; and countenance expressive of anxiety. A careful examination was instituted for latent or concealed hemorrhage, but the uterus was found to be well contracted, and no more than the usual amount of blood had been lost. To me, the great and positively alarming constitutional depression was unaccountable. I could not think that her labor had been sufficiently prolonged or severe to have so nearly exhausted her, unaided by other, and, to me, mysterious, causes. Stimulants were administered.

At 2 A.M., her condition had manifestly improved. We then directed quinia sulphas, gr. ij, every two hours, and left her for the remainder of the night. I visited her at 10 A.M. She had not slept; suffered no pain, but was very restless; her pulse was 120, though possessing more strength than it did when we left her, eight hours previously; her skin was naturally warm. I ordered the quinia sulphas to be omitted, and directed morph. sulphas, gr. j, every two hours, until sleep was obtained.

9th, 11 A.M. Had slept considerably. Pulse 100; urine had been freely voided. The anodyne to be used *pro re nata.* 7 P.M. Hastily summoned. Dr. ORRIN SMITH in council. She was much agitated; pulse 120, though weak; surface moderately warm; countenance anxious; suffering much pain in the region of the uterus; abdomen very tympanitic; lochia arrested; in fact, all the symptoms of puerperal metritis. We ordered opium, gr. 1½, every two hours; tinc. verat. vir., gtt. v, every fourth hour.

10th, 8 A.M. Dr. O. SMITH in council. Suffering but little pain; abdomen more tympanitic; great tenderness in the utering region; pulse 100. Treatment continued. 6 P.M. Pulse 90, which was the only appreciable change. Lessened the quantity of veratrum, and ordered hops as a topical application.

11th and 12th. The symptoms have not sufficiently varied from the date of the last report to be worthy of note. The same treatment continued with slight modification.

13th, 5 P.M. Dr. N. S. DAVIS in council. She is much agitated about the tympanitic shape of the abdomen, and the absence of the lochia; pulse 115; not much pain, though quite tender over the womb. Hyd. chlo. mite, gr. ij, opium pulv., gr. $1\frac{1}{2}$, every two hours; tinc. verat. vir. in small doses; hops externally; and additional nourishment.

14th. Her bowels have been freely moved, for the first time since her confinement. The discharges were excessively fœtid. Tympanitis almost subsided, no pain, and but little tenderness.

15th, 16th, and 17th. Apparently convalescent. Requires but little medicine.

18th. Very feeble; pulse 120; skin cold; perspiring freely. Has been in this condition much of the night and morning. Quinia sulphas was ordered.

19th, 5 P.M. Dr. DAVIS in council. Patient is unable to control the action of the sphincters; bowels relaxed. Nitric acid, f. ʒj, tinc. opii., f. ʒij, strychnia, gr. j, aquæ distillata, f. ℥iij, of which a teaspoonful shall be taken every fourth hour; quinia sulphas, gr. ij, every second hour.

20th, 7 A.M. Twelfth day after the birth of the child. Almost *in articulo mortis;* uterine hemorrhage commenced at 4 A.M., and continued moderately until about 6 o'clock, when it became terrific in its flow. She is now speechless; involuntary discharges; bathed in cold and clammy perspiration; features very much shrunken; and pulse, at the wrist, scarcely perceptible. She seems to have passed a vast deal of blood, though the syncope has mainly checked its further issue. The head was lowered, lower extremities elevated, a compress, saturated with ice water, was applied to the vulva and retained by appropriate appliances. The effect was the tamponization of the vagina by large coagula, which remained almost two days. Opii, plum., acetas, quinia sulphas, brandy, &c., were given. Reaction was not established until 5 P.M., and at this hour she appears almost exsanguine.

21st, 22d, and 23d. Slowly improves under tonics and stimulants. She claims that there is no discharge from the vagina of any nature.

24th, 6 P.M. 16th day after confinement. Was feeling exceedingly well until about 5 P.M. No symptom, except feebleness, of which she could justly complain. At this hour, she felt cold and asked for a blanket, and a little later, the bleeding had returned, with all the violence that had characterized it five days previously. When I reached her residence, the immediate friends had gathered around her, and all believed that she was dying. She was unconscious, and I had little thought that she would again rally. The treatment adapted to such conditions was pursued.

25th, 10 A.M. Some little warmth of skin; feeble and frequent pulse; was unable to speak until this morning; no further hemorrhage; no pain. I ordered ferri per-sulphas, vin. ergotæ, and quinia sulphas.

26th, 1 P.M. Dr. BYFORD in council. Patient looks as if she were bloodless; pulse 125. A vaginal examination is made. Os uteri very pabulous; some offensive discharge, nothing else determined by it. A very careful examination over the uterus, demonstrates, quite clearly, its boundaries. It is, evidently, much larger than it should be on the nineteenth day after confinement. Prognosis very unfavorable, more in consequence of the prostration which is dependent mainly upon the great loss of blood, than aught else. Iron, ergot, vaginal injections, counter-irritants, with nourishment, given in a methodical manner, constitute the treatment.

29th. Has improved. Omit the vaginal injections, for fear that they might act as a provocative of the hemorrhage.

June 9th. Hemorrhage recurred this morning, and continued for a few hours. It was small in amount, and did not very materially reduce her strength. From this date, she continued to improve, and, after the expiration of several months, was fully restored to health.

The variety of uterine hemorrhage occurring in the case just described, is alluded to, by authors, under the appellation of "secondary *post partum* hemorrhage." The cases no doubt are exceptional, where the uterus has contracted subsequently to the birth of the child, and become relaxed, resulting in an indefinite amount of bleeding.

Prof. BEDFORD says, that it may occur at any time after delivery, from two hours to two or three weeks. Other authorities could be cited, who mention instances where it has taken place as late as two or three months after the expulsion of the fœtus, but I believe the best authors are not inclined to associate the latter class of cases with delivery, but deem that their more appropriate place is under the title of passive hemorrhages. It is proper to remember, also, that there are instances recorded where, it is said, hemorrhage has taken place from a well contracted uterus. VELPEAU, in his obstetrical work, page 591, says, "I have seen, on two occasions, hemorrhage after the delivery of the placenta, although the womb was well contracted on itself; the labor had been terminated four hours in one case, and seven in the other. It is, besides, an accident, not uncommon after the first twenty-four hours." GOOCH gives credence to the statement, and INGLEBY cites cases to prove the same doctrine. ROBERTSON opposes it, and maintains that at least the fundus of the uterus must have been relaxed. Mad. BOIVIN believes, that hemorrhage and contraction of the uterus are incompatible, and insists that the blood which escapes from a contracted womb does not flow from the uterine vessels, but from the placenta, as from a compressed sponge.

As to the more prominent causes of secondary *post partum* hemorrhage, authors seem to be agreed. The retention of some portion of the placenta or membranes, is one of the most frequent causes. The recorded experience of DE LA MOTTE, LEROUX, and DELEURYE should be sufficient to warn the obstetrician of the necessity of exercising due vigilance, in the removal of detached portions of the secundines. If it is decided to introduce the hand into the uterine cavity in such a case, as advised by VELPEAU and others, it would be well to remember that BLUNDELL and INGLEBY question its propriety.

Coagula of blood in the uterus, is also mentioned as a cause. Madame LA CHAPELLE and COLLINS have both recorded examples of bleeding commencing eight and ten days after delivery, occasioned by coagula. The removal of the coagula by the hand would seem to be indicated.

Dr. MCCLINTOCK, of Dublin, and Prof. BEDFORD, of New York, also give much prominence to two other causes. The language of the latter is:—"The bleeding may be the result of an atonic condition of the uterus, not amounting to positive inertia, but occasioning a partial flaccid state of the organ giving rise to hemorrhage." When owing to such a cause, the flow will be moderate. No heroic treatment will be demanded, but almost always, ergot may be relied upon to accomplish the arrest of the bleeding. One author advises, under such circumstances, that the ergot shall be assisted by the injection, into the rectum, of cold water twice daily. The other cause assigned by the authorities last quoted is, congestion of the organ in plethoric women, unattended by inertia, and advise the abstraction of blood from the arm, administration of saline cathartics, and an abstemious diet.

The cause of the sudden and frightful hemorrhage of the case under my care, and of which I have given the clinical history, was not, in my opinion, attributable to the retention of any portion of the placenta or membranes, or to the existence of coagulated blood. The uterus was well contracted after delivery. What effect the metritis may have had in causing subsequent relaxation, which resulted in the several attacks of such terrible secondary hemorrhage, I leave for others to judge.

